# Using Bayesian event probabilities for monitoring clinical quality assurance

**DOI:** 10.1093/bjsopen/zrag082

**Published:** 2026-07-20

**Authors:** Edward H Livingston, Ami Hayashi, Kyle D Klingbeil, Anne Y Lin

**Affiliations:** Department of Surgery, UCLA School of Medicine, Los Angeles, California, USA; Department of Surgery, UCLA School of Medicine, Los Angeles, California, USA; Department of Surgery, UCLA School of Medicine, Los Angeles, California, USA; Department of Surgery, UCLA School of Medicine, Los Angeles, California, USA

**Keywords:** quality of care, monitoring surgical outcomes, quality metrics, monitoring postoperative complications

## Abstract

**Background:**

Current systems used to monitor clinical quality metrics are expensive and use, not used outdated statistical methodologies. The aim of this study was to develop and test a Bayesian approach for continuous monitoring of clinical outcomes using information easily obtained from the electronic medical record.

**Methods:**

An algorithm was developed with the assistance of artificial intelligence to extract surgical outcomes data from electronic medical records and to then calculate the posterior probability distribution for complication occurrence. A test data set was created from a retrospective analysis of the complete medical records of patients receiving surgical care at UCLA Health between 1 January 2016 and 22 September 2024. The 20 most performed inpatient colorectal operations were considered the index cases for the ‘major colorectal procedures’ group. Complications were identified by diagnostic codes in the medical record and attributed to the index procedure if diagnosed within 1 year after surgery. Bayesian regression was used to determine the posterior probability of complication occurrence for comparison with pre-established acceptability thresholds. A > 50% probability of complication occurrence exceeding the threshold would trigger a quality assurance review for that complication during the period of interest.

**Results:**

There were 747 patients who underwent major colorectal operations. Postoperative ileus (7.4%) and anastomotic leak (3.9%) were the most common complications, whereas postoperative bleeding was rare (0.3%). Of the ten complications tracked, the number triggered for chart review ranged from a low of two in 2016 to six in 2022. The largest number of excess complications triggering review was three in any year.

**Conclusion:**

A system for real-time monitoring of complications was modelled from retrospective data. It successfully identified time periods in which the probability distribution for experiencing complications exceeded pre-established thresholds, identifying a need for chart review. A Bayesian event probability tracker will be inexpensive to implement, could track clinical outcomes in near real-time (facilitating data review whenever it is desired), and is statistically valid.

## Teaser

Current systems used to monitor surgical quality assurance are resource intensive, provide analyses long after adverse events occur, and use statistical methods that are problematic. This paper proposes new methodology that capitalizes on modern integrated health system electronic health records that can monitor outcomes in near real time. Bayesian statistics are used to provide probability distributions for complication occurrence relative to accepted benchmarks. The system would cost very little to operate.

## Introduction

Postoperative complications remain a major source of patient harm, prolonged hospitalization, readmission, and increased healthcare costs. Many of these adverse events occur or are identified after discharge, making timely identification a significant challenge for hospital quality improvement. Current surveillance systems that rely on manual chart review or delayed administrative reporting often fail to detect issues until months after the complication has occurred, frequently well past the point at which intervention could benefit current patients^1^. As a result, quality assurance (QA) processes tend to be reactive rather than proactive, costly, and have less than ideal impact on patient safety.

Effective QA requires real-time feedback that is clinically actionable and financially sustainable. Existing US-based national and institutional monitoring systems such as the National Surgical Quality Improvement Program (NSQIP), the Healthcare Effectiveness Data and Information Set (HEDIS), the Center for Medicare and Medicaid Service (CMS) Meaningful Measures Framework, and the Joint Commission’s ORYX^®^ system provide robust outcome tracking but require substantial financial and personnel investment. These systems deliver information on quality outcomes with considerable delay, limiting their utility for near-real-time intervention^2–6^. These systems provide robust, risk-adjusted outcome tracking and inter-hospital benchmarking (NSQIP), performance measurement for preventive care (HEDIS), quality metric reporting (CMS Meaningful Measures), and standardized outcome monitoring (Joint Commission ORYX^®^), but all rely on periodic data submission, manual abstraction, or delayed reporting, limiting their ability to detect emerging issues in near real-time.

To address these limitations, an automated, electronic medical record (EMR)-derived Bayesian monitoring algorithm is needed that can continuously collect information about adverse events occurring after surgery and update the probability of postoperative complications. By extracting data directly from the EMR and using Bayesian probability to determine when complication rates exceed predefined thresholds, such a system will be able to deliver real-time, clinically relevant alerts to prompt further chart review. The aims of this study were to test the algorithms needed for such a system with retrospective data on colorectal surgical outcomes within a large academic health system and to generate benchmark values for expected complication rates that can occur after colorectal surgery.

## Methods

This cohort study of patients who underwent colorectal surgery at UCLA Health was performed on a database created by a medical record search of care delivered between 1 January 2016 and 22 September 2024. The analysis of these data was approved and the requirement for informed consent waived by the UCLA Institutional Review Board (UCLA IRB-24-1261). Inclusion and exclusion criteria for database creation are provided in the *supplementary methods*.

A search of the 20 most common procedures performed on patients in the database was conducted based on Current Procedural Terminology (CPT) codes (*Table S1*). Those associated with major colorectal operations (CPT 44XXX, except laparoscopic appendicectomy 44970) were aggregated into a single category referred to as ‘major colorectal procedures’. Complications commonly associated with colorectal procedures were identified by expert opinion and found in the medical records by their International Classification of Diseases, Tenth Revision (ICD-10) codes^7^. The ten complications and their ICD-10 codes selected for analysis are shown in the *supplementary methods*. If the complication codes appeared in the medical record within 1 year of an operation, it was assumed that the complication was associated with the index procedure. Procedures and the complications associated with them were stratified by calendar year. The primary outcome was the Bayesian posterior distribution of the probability of experiencing any of the designated complications in any given year. The thresholds of acceptability for complication rates were determined as the mean complication rate for the entire data set.

### Statistical analysis

#### Conceptual overview of Bayesian monitoring

Traditional quality monitoring often uses fixed thresholds and simple percentages to identify high complication rates, but these approaches can be overly rigid or miss gradual changes. Bayesian methods treat the true complication rate as an unknown probability that can be periodically updated with new data, producing a full probability distribution (posterior) rather than a single *P* value. This lets providers answer clinically relevant questions, such as ‘What is the probability that the current rate exceeds an acceptable benchmark?’ In the present study, Bayesian binomial models for complication incidence (updating beliefs yearly) and exponential models for time-to-complication were applied, using non-informative priors to let the data drive conclusions while providing uncertainty estimates (credible intervals) that narrow as more information accumulates^8^.

#### Bayesian model for complication incidence

The incidence of each complication within 1 year after the procedure was modelled year-by-year using a Bayesian binomial framework to estimate the probability *p* of a patient experiencing the complication. The binomial distribution is characterized by the number of complications (*k* of *n* procedures) each year as binomial, with the unknown rate *p* following a beta distribution. For each complication type and year, *k* is the number of unique affected patients and *n* is the total number of unique patients undergoing procedures in that year. The likelihood is binomial: *k* ∼ Binomial(*n*,*p*). A non-informative Jeffreys prior was placed on *p*: *p*  *∼* Beta(α = 0.5, β = 0.5). This prior was chosen for its objectivity and invariance properties, allowing the data to primarily influence the posterior while providing a reference distribution for proportions. The same prior was used for each year of the analysis.

The posterior distribution for *p* is [*p* | *k*,] *n* ∼ Beta(α + *k*, β + *n* – *k*). This updating process allows the estimate to incorporate all previous years’ data naturally, without treating each year as completely independent.

#### Bayesian model for time to complication

Time to complication was modelled as an exponential distribution with non-informative Gamma priors (α = 0.001, β = 0.001). Posterior sampling (10 000 iterations) estimated the mean θ = 1/λ, median, and 95% credible interval, stratified by encounter type (≥ 2 cases required).

#### Posterior summaries and inference

Posterior summaries and inference values were calculated as detailed below:

- Point estimates: the posterior mean was calculated as $\hat{\;p}=\frac{\alpha +k}{\alpha +\beta +n}$
- Credible intervals: the 95% credible intervals were derived as the 2.5th and 97.5th quantiles of the posterior Beta distribution as: [qbeta(0.025,α+k,β+n−k),qbeta  (0.975,α+k,β+n−k)]
- Comparison to overall rate: for each year and complication, the overall aggregated complication rate (across all years) was computed as a benchmark $p_{\mathrm{overall}}=\frac{\sum k}{\sum n}$. The posterior probability that the year’s rate exceeded this benchmark was calculated as $\mathrm{Pr}(\;p>\;p_{\mathrm{overall}}|k,n)=1-\mathrm{pbeta}$  $\left(\;p_{\mathrm{overall}},\alpha +k,\beta +n-k\right)$. Years where this probability exceeded 0.5, 0.8, and 0.95 were flagged in individual analyses as having elevated risk relative to the aggregate.

#### Posterior predictive check of model calibration

To assess the adequacy and calibration of the Bayesian Beta–Binomial model for complication incidence rates, posterior predictive checks were performed. For each complication and year, 10 000 replicated complication counts (*k*_rep) were simulated from the cumulative posterior distribution: *p*_rep ∼ Beta(α_year, β_year), then *k*_rep ∼ Binomial(*n*_year, *p*_rep). The observed complication count (*k*) was compared to the distribution of *k*_rep by computing the Bayesian *P* value, which is the proportion of replicates where the absolute deviation from the predictive mean was at least as large as the observed deviation (two-tailed, deviation-based). It was also determined whether the observed *k* fell within the 95% posterior predictive interval of *k*_rep and the mean and variance of replicated counts *versus* observed.

#### Sensitivity analyses for the Gamma prior in the exponential time-to-complication model

To estimate mean time from surgery to complication diagnosis, waiting times were modelled as an exponential (constant hazard rate), starting with a very vague Gamma prior on the rate λ. The exponential distribution was used to model time-to-complication (in days) from the index colorectal procedure to diagnosis of the complication, restricted to events occurring within 1 year after the procedure. The rate parameter λ was assigned a non-informative Gamma (α = 0.001, β = 0.001) prior in the primary analysis, yielding posterior estimates of mean time θ = 1/λ. Analyses were stratified by complication type and year (group = Complication_Year), and only groups with two or more timed events were included (55 groups, total 897 timed events). Sensitivity analyses were performed by re-estimating the model under five alternative Gamma priors on λ, as follows:

- original (very vague): Gamma(0.001, 0.001)
- vague alternative: Gamma(0.01, 0.01)
- weakly informative (mean λ ≈ 1, θ ≈ 1 day): Gamma(1, 1)
- moderately informative (mean λ = 4, θ = 0.25 days): Gamma(2, 0.5)
- stronger informative (mean λ = 5, θ = 0.2 days): Gamma(5, 1).

For each prior, Markov chain Monte Carlo sampling was conducted using Stan (four chains, 10 000 iterations per chain, adapt_delta = 0.95). Posterior summaries for θ (mean, median, and 95% credible intervals) were extracted for each group. Results were combined across priors and visualized to assess the stability of estimates. All computations were performed in R version 4.5.0 (R Foundation for Statistical Computing, Vienna, Austria) with the rstan package.

#### Benchmarks

The mean rate of complications for the entire database for each complication was used as the benchmark value for expected complication rates in the study institution. The reasonableness of these values was assumed based on literature review for reports of colorectal surgery complication rates. All analyses were performed using R version 4.5.0 with Grok assistance for code development.

## Results

Patient characteristics are summarized in *Table 1*. There were 747 patients included in the study, with a mean age of 63 years and an equal sex distribution. Most of the patients were White, with fewer patients who identified as Hispanic, Black, or Asian. White patients were somewhat over-represented (46%) compared with the Los Angeles population (30%) and Hispanics were under-represented (19% *versus* 48%), with Black and Asian patients being approximately proportional to the region’s demographic mix. Hypertension, diabetes, and renal insufficiency were present in 21%, 19% and 10% of patients, respectively (*Table 1*).

**Table 1:** Baseline characteristics of the 747 patients with major colorectal operations included in this analysis

Age (years), mean(s.d.)	63(18)
**Sex**	
Female	385 (51.5%)
Male	362 (48.5%)
**Race**	
American Indian or Alaska Native	4 (0.5%)
Asian	72 (9.6%)
Black or African American	70 (9.4%)
Do not identify with race	27 (3.6%)
Middle Eastern or North African	25 (3.3%)
Multiple races	17 (2.3%)
Native Hawaiian or other Pacific Islander	4 (0.5%)
Other	135 (18.1%)
Patient refused	28 (3.7%)
Unknown	24 (3.2%)
White or Caucasian	341 (45.6%)
**Ethnicity**	
Chose not to answer	37 (5.0%)
Hispanic or Latino/a	117 (15.7%)
Mexican, Mexican American, or Chicano/a	11 (1.5%)
Not Hispanic or Latino/a	573 (76.7%)
Other Hispanic, Latino/a, or Spanish origin	8 (1.1%)
Unknown	1 (0.1%)
**Procedure descriptions**	
Close enterostomy, resection + anastomosis	101 (13.5%)
Close enterostomy, resection + colorectal anastomosis	20 (2.7%)
Laparoscopy, surgical colostomy	51 (6.8%)
Laparoscopy, surgical ileo-/jejunostomy	63 (8.4%)
Laparoscopy, surgery, colectomy, partial, with anastomosis	68 (9.1%)
Laparoscopy, surgery, colectomy, with anastomosis	65 (8.7%)
Laparoscopy, surgery, colectomy, with removal of terminal ileum	71 (9.5%)
Part removal of the colon with anastomosis	99 (13.2%)
Part removal of the colon with coloproctostomy	46 (6.2%)
Part removal of the colon with end colostomy	41 (5.5%)
Removal of the colon and terminal ileum with ileocolostomy	122 (16.3%)
Diabetes	142 (19.0%)
Hypertension	158 (21.2%)
Smoking	30 (4.0%)
Renal insufficiency	77 (10.3%)
COPD	24 (3.2%)
CHF	45 (6.0%)
Malignant neoplasm of the colon	151 (20.2%)
Diverticulitis	68 (9.1%)

Values are *n* (%) unless otherwise stated. s.d., standard deviation; COPD, chronic obstructive pulmonary disease; CHF, congestive heart failure.

The spectrum of complications observed for all the years of analysis combined and previously reported benchmark values reported in the literature are presented in *Table 2*. There were 747 major colorectal resections performed between 2016 and 2024, with 108 complications yielding an overall complication rate of 15%. Postoperative ileus (7.4%) and anastomotic leak (3.9%) were the most common, whereas postoperative bleeding was rare (0.3%). Literature-based benchmarks were sought by examining articles reviewing colorectal surgery complications. Ranges for complication rates reported in these reviews are provided if numerical results were given. Complication rates in the present series were at the low end or below the reported literature benchmarks when they were available.

**Table 2:** Complications (*n* = 151) occurring in the 747 patients included in this analysis

Complication	No. of patients affected (%)	Literature benchmark
Anastomotic leak	29 (3.9%)	2–30%^9–12^
Bowel obstruction	9 (1.2%)	NA^12^
Hernia	15 (2.0%)	NA^9,12^
Postoperative bleeding/haemorrhage	2 (0.3%)	NA^9,13^
Postoperative ileus	55 (7.4%)	7.5–30%^9,12,14^
Postoperative pneumonia	15 (2.0%)	6–20%^9,11^
Pulmonary embolism	16 (2.1%)	NA^9^
Surgical site infection	10 (1.3%)	2–26%^9,11,14–16^

NA, publications that described the complications but did not provide a numerical range that could be used as a benchmark value.

The number of procedures and complications occurring in each year of the study are presented in *Table 3*. In addition, *Table 3* presents the number of patients who would require complete chart review if there was a greater than 50%, 80%, or 95% probability that the observed complication rate exceeded the threshold value for the individual complication rates for each type of complication in each year. All patients in each category of complication category are flagged for review, whereas none of the patients in the categories of complications that were not outliers for that year were flagged. Progressively fewer cases need review with greater probabilities of exceeding the threshold acceptable complication rates. At 50%, nearly all complications are reviewed. At the normally accepted 95% level for statistical significance, almost no cases need to be reviewed. At an 80% probability, a manageable number of cases would be reviewed (*Table 3*).

**Table 3:** Number of major colorectal resection procedures per year, complications, and patients requiring chart review at different thresholds for the probability of annual complication rates exceeding accepted benchmarks

Year	No. of procedures per year	No. of patients with complications	No. of patients flagged for review		
			50% Threshold	80% Threshold	95% Threshold
2016	112	13	4	4	0
2017	99	16	16	13	0
2018	86	12	11	6	0
2019	101	15	12	4	0
2020	92	12	11	6	0
2021	72	12	6	5	4
2022	55	10	10	8	0
2023	75	11	7	6	4
2024	55	7	6	4	0

*Table 4* presents the mean time to the diagnosis of the complications. None of these complications were noted in the discharge records for the index admissions for the operations reviewed; all were encountered in subsequent hospitalizations or clinic visits. Analysis of hospital readmissions found that 34 complication diagnoses were present on admission, 64 were listed as the primary diagnosis, 17 were the admission diagnosis, and 89 were the final hospital diagnosis.

**Table 4:** Time to complication

Complication	No. of patients	Time to complication (days)		95% Credible interval
		Mean	Median	
Postoperative ileus	55	30.3	29.9	23.1, 39.5
Surgical site infection	10	35.4	32.9	18.4, 66.9
Postoperative pneumonia	15	42.6	40.7	25.6, 71.0
Bowel obstruction	9	18.1	16.7	9.1, 35.3
Pulmonary embolism	16	35.6	34.1	21.6, 58.0
Anastomotic leak	29	27.7	27.0	19.3, 40.0
Postoperative bleeding/haemorrhage	2	44.1	25.9	7.8, 181.4
Hernia	15	42.7	40.7	25.9, 71.4

**Fig. 1:**
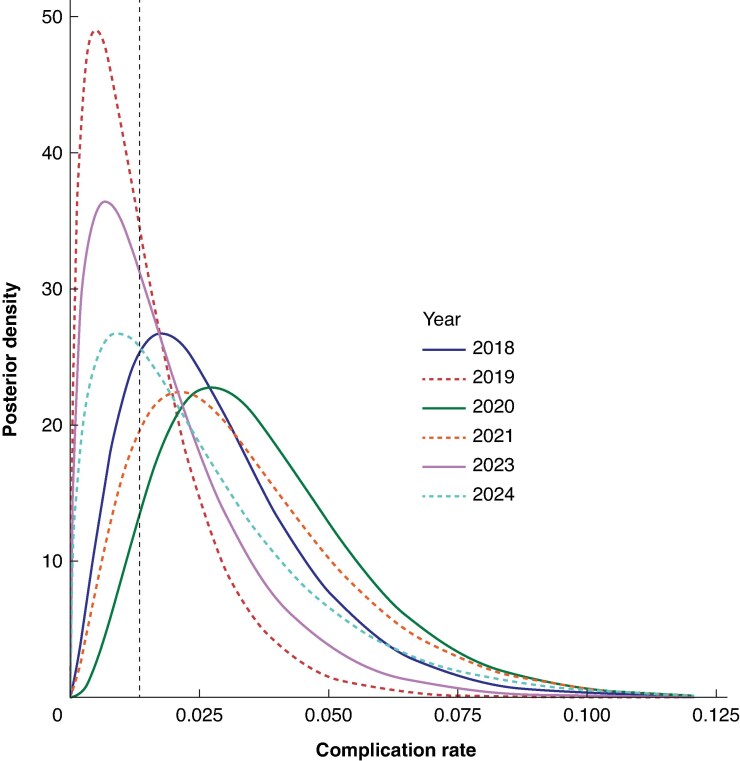
Typical probability distribution for Bayes analysis of clinical outcome data (surgical site infection)

**Fig. 2:**
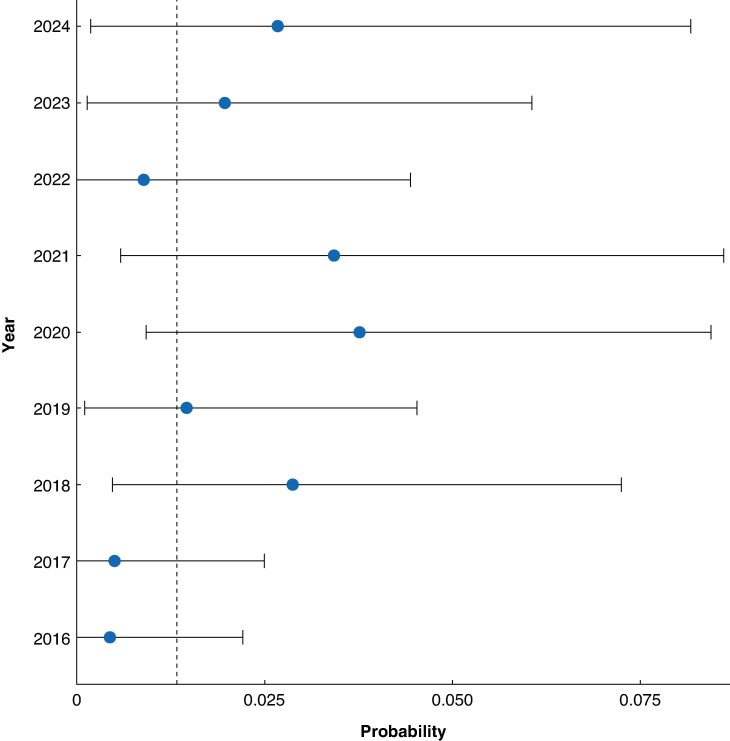
Probability of 90-day surgical site infection complication by year Error bars indicate 95% credible intervals.

*Figure 1* shows a typical probability distribution. A stacked line representation of annual complication rate mean values and 95% credible intervals is shown in *Fig. 2*. *Table 5* summarizes the years in which there was a > 50% probability that complications exceeded pre-established threshold values indicating that a QA review was warranted. The number of complication diagnoses triggered for review ranged from two in 2016 to six in 2022. *Table 6* presents the years in which complication rates had a > 50% probability of exceeding the complication rate threshold and the number of excess patients above the threshold that was responsible for the increase. The largest number of excess patients with complications in any given year was three.

**Table 5:** Years and complications that would have been flagged for review based on having a > 50% probability of the complication incidence exceeding the acceptable threshold

Complication	Year	Probability*
Postoperative ileus	2017	0.85
Postoperative ileus	2020	0.7
Postoperative ileus	2022	0.84
Postoperative ileus	2024	0.71
Surgical site infection	2018	0.81
Surgical site infection	2020	0.93
Surgical site infection	2021	0.86
Surgical site infection	2023	0.57
Surgical site infection	2024	0.69
Postoperative pneumonia	2017	0.92
Postoperative pneumonia	2018	0.63
Postoperative pneumonia	2019	0.91
Postoperative pneumonia	2022	0.82
Bowel obstruction	2018	0.56
Bowel obstruction	2019	0.79
Bowel obstruction	2020	0.82
Bowel obstruction	2021	0.63
Bowel obstruction	2022	0.72
Bowel obstruction	2023	0.88
Pulmonary embolism	2017	0.51
Pulmonary embolism	2018	0.93
Pulmonary embolism	2021	0.96
Anastomotic leak	2017	0.74
Anastomotic leak	2018	0.67
Anastomotic leak	2019	0.73
Anastomotic leak	2020	0.62
Anastomotic leak	2022	0.51
Anastomotic leak	2024	0.9
Postoperative bleeding/haemorrhage	2016	0.9
Postoperative bleeding/haemorrhage	2020	0.92
Postoperative bleeding/haemorrhage	2021	0.53
Postoperative bleeding/haemorrhage	2022	0.59
Postoperative bleeding/haemorrhage	2023	0.53
Postoperative bleeding/haemorrhage	2024	0.59
Hernia	2016	0.88
Hernia	2017	0.55
Hernia	2019	0.54
Hernia	2022	0.53
Hernia	2023	0.97
Hernia	2024	0.53

^*^The probability of experiencing complication rates greater than the threshold for that year.

**Table 6:** Complication and year when the probability of a complication was ≥ 50% than the pre-established threshold

Complication	Year	No. of required reductions*
Postoperative ileus	2017	3
Postoperative ileus	2020	2
Postoperative ileus	2022	3
Postoperative ileus	2024	2
Surgical site infection	2018	2
Surgical site infection	2020	2
Surgical site infection	2021	2
Surgical site infection	2023	1
Surgical site infection	2024	1
Postoperative pneumonia	2017	3
Postoperative pneumonia	2018	1
Postoperative pneumonia	2019	3
Postoperative pneumonia	2022	2
Bowel obstruction	2018	1
Bowel obstruction	2019	1
Bowel obstruction	2020	2
Bowel obstruction	2021	1
Bowel obstruction	2022	1
Bowel obstruction	2023	2
Pulmonary embolism	2017	1
Pulmonary embolism	2018	3
Pulmonary embolism	2021	3
Anastomotic leak	2017	2
Anastomotic leak	2018	1
Anastomotic leak	2019	2
Anastomotic leak	2020	1
Anastomotic leak	2022	1
Anastomotic leak	2024	3
Postoperative bleeding/haemorrhage	2016	1
Postoperative bleeding/haemorrhage	2020	1
Postoperative bleeding/haemorrhage	2021	NA
Postoperative bleeding/haemorrhage	2022	NA
Postoperative bleeding/haemorrhage	2023	NA
Postoperative bleeding/haemorrhage	2024	NA
Hernia	2016	2
Hernia	2017	1
Hernia	2019	1
Hernia	2022	1
Hernia	2023	3
Hernia	2024	1

^*^How many fewer patients with complications would be required for the probability of a complication to be < 50% below the threshold (that is, the excess number of patients with complications).

Bayesian analysis of complication rates following major colorectal procedures from 2016 to 2024 revealed varying posterior mean rates across the ten monitored complications, with sequential updating incorporating cumulative data over time. Overall posterior estimates (based on 2024 cumulatives) ranged from very low for rare events like deep vein thrombosis (mean rate 0.0011; 95% credible interval 0.0000 to 0.0042) and peristomal skin irritation (mean rate 0.0011; 95% credible interval 0.0000 to 0.0042) to higher for common issues such as postoperative ileus (mean rate 0.1455; 95% credible interval 0.1230 to 0.1695). Anastomotic leak stabilized around a mean rate of 0.0807 (95% credible interval 0.0636 to 0.0995), whereas the mean rate of surgical site infection showed a slight increase then stabilization at 0.0205 (95% credible interval 0.0122 to 0.0308). The mean rate of postoperative bleeding/haemorrhage remained low at 0.0045 (95% credible interval 0.0012 to 0.0099). Bowel obstruction and hernia rates were moderate at 0.0318 (95% credible interval 0.0213 to 0.0444) and 0.0727 (95% credible interval 0.0565 to 0.0908), respectively. The mean rates of pulmonary embolism and postoperative pneumonia were 0.0432 (95% credible interval 0.0308 to 0.0576) and 0.0534 (95% credible interval 0.0395 to 0.0692), respectively. Across complications, credible intervals narrowed over time due to accumulating data, with no dramatic year-over-year shifts, suggesting stable institutional performance (*Table S2*).

The results of posterior predictive checks indicated good model fit overall: Bayesian *P* values ranged from 0.120 to 0.880 (non-extreme < 0.050 or > 0.950) and observed *k* fell within the 95% predictive interval in 92% of year–complication pairs (*Table S3*). This supports that the model adequately captures the data-generating process without substantial misfit or miscalibration. Model adequacy was further confirmed by posterior predictive checks, which showed excellent calibration with Bayesian *P* values consistently in the 0.100–0.900 range and observed counts falling within predictive intervals in 92% of cases (*Table S3*).

The sensitivity analyses for the Gamma prior in the exponential time-to-complication model showed that for groups with ≥ 20 events (for example, postoperative ileus, anastomotic leak, hernia, pulmonary embolism), posterior mean θ varied by < 15% across priors, indicating strong robustness to prior choice. Smaller groups (< 10 events) showed greater sensitivity, with stronger priors shrinking θ towards lower values (consistent with prior means). Overall, these findings demonstrate stability of time-to-event estimates in well-powered strata, although sparse data warrant caution in interpretation for low-event complications (*Table S4* and *Fig. S1*).

To determine how these analyses would be used in clinical practice, postoperative pneumonias for 2017 were examined in detail. In 2017, the probability of exceeding the threshold postoperative pneumonia rate of 2% (*Table 3*) was 92% (*Table 5*). Examining the data underlying *Table 6* revealed that, for 2017, there were 99 major colorectal surgeries performed with four (4.0%) patients experiencing postoperative pneumonia. To fall below the 2% threshold, there should have been three fewer cases of pneumonia. Chart review of these four cases of pneumonia revealed that two of them occurred in patients with widely metastatic cancer, one of whom developed severe cardiac arrhythmias unrelated to their surgery and had pneumonia secondary to cardiac disability. A third patient with pneumonia who underwent an operation for multiply recurrent rectal cancer was in a debilitated state and had an anastomotic leak attributable to many previous operations; this patient developed pneumonia while recovering from the leak. The fourth patient had dementia and known recurrent aspiration events from a swallowing disorder. This review found no quality-of-care issues accounting for the higher-than-expected postoperative incidence of pneumonia.

## Discussion

To avoid future harm to patients, it is imperative to promptly identify clinical care deficiencies. Most QA systems used today are retrospective, relying on data abstraction, processing, and analysis, followed by dissemination of reports to providers. This process may uncover problems many months after they occur. To avoid future harm to patients, QA data should be assessed as close to the care event as possible. Ideally, clinical care outcomes should be monitored prospectively and acted upon immediately^17–23^.

The present retrospective analysis of EMR data showed the feasibility of a prospective system of frequent complication rate monitoring using Bayesian statistical methods. By adopting a Bayesian binomial framework with non-informative priors, posterior distributions for complication probabilities were estimated and direct probabilities that annual rates exceed institution-derived benchmarks were calculated. At an 80% posterior probability that observed complication rates exceed pre-established threshold values, a manageable number of cases warranted review, whereas a conventional 95% threshold (analogous to frequentist significance) flagged almost none, highlighting how frequentist fixed-α rules (that is, *P* < 0.05) can be overly conservative in repeated monitoring^24,25^.

Bayesian statistical analysis directly yields the probability that an event occurs (for example, that the true complication rate exceeds an acceptable threshold), which is the information clinicians most often seek in quality assurance. In contrast, *P* values from frequentist methods estimate the probability of observing the data (or more extreme data) assuming no difference exists (the null hypothesis); they do not quantify the probability that the complication rate is truly elevated. Bayesian methods start with any available information about the phenomenon (the prior distribution), update it with the observed data (the likelihood), and produce the posterior distribution. This posterior reflects the updated belief about true complication rates, allowing direct calculation of probabilities such as ‘There is an 80% posterior probability that the rate exceeds the benchmark’. Uncertainty in these estimates is summarized by a 95% credible interval, which is the range containing the true value with 95% posterior probability, given the data and prior (unlike frequentist confidence intervals, which are often misinterpreted as having this same meaning). Whereas frequentist statistics treat each data set as completely independent (assuming parameters are fixed unknowns) and penalize repeated analyses through inflated false-positive risks, Bayesian methods naturally incorporate previous information and support frequent updating, leading to progressively refined probability estimates. Thus, Bayesian approaches analyse frequently sampled QA data more efficiently and intuitively^24–27^.

Frequentist statistical methods dominate current QA monitoring but are poorly suited to true continuous or repeated surveillance of surgical outcomes. These approaches assume fixed, independent samples and disallow incorporation of previous knowledge, treating each analysis in isolation. The *P* values derived from frequentist statistics do not determine the probability that a complication rate is or is not acceptable. Rather, they represent the probability of observing data in a study that was at least as extreme as what was actually seen, assuming there is no actual effect (the null hypothesis). *P* values do not quantify the probability that a complication rate exceeds a threshold of concern, the main question in QA. This direct probabilistic interpretation is a key strength of Bayesian analysis^27–30^.

Although frequentist methods for sequential surveillance do exist, such as cumulative sum (CUSUM) charts, risk-adjusted CUSUM, sequential probability ratio tests, and group-sequential designs with multiplicity-adjusted boundaries (for example, Pocock or O'Brien-Fleming), these approaches typically provide binary ‘alarm’ signals based on crossing predefined control limits and require careful calibration of error rates across the monitoring horizon to avoid excessive false positives. In contrast, the current Bayesian framework naturally supports ongoing, sequential updating of the posterior distribution without ad hoc multiplicity adjustments, directly computes the posterior probability that the true rate exceeds a benchmark (for example, 80% threshold for review), and provides an intuitive probabilistic summary of belief in elevated risk.

With frequentist methods, each new analysis is treated independently, so repeated testing without adjustment inflates the overall false-positive rate, increasing the chance of falsely identifying outliers and labelling acceptable care as problematic. For example, even a provider delivering consistently acceptable performance may be flagged for review approximately 5% of the time per assessment purely due to random sampling variation under fixed α-level rules^31^. Over multiple assessments, the cumulative risk of at least one false alarm grows substantially. Observed/expected ratios and process control charts suffer from similar multiple-testing issues, and their statistical analyses often assume independence of each analytic sample, an assumption violated by ongoing monitoring^32–35^.

Unlike NSQIP or CMS programmes, which require manual extraction of medical record data, the method described in this study uses routinely available data for near-real-time complication monitoring. Because the proposed system avoids the need to manually extract data, it should cost little to implement. Costs associated with QA data collection are not trivial. In one large health system, €5.2 million (US$5.6 million) was spent in 1 year on preparing data and paying vendor fees for quality assurance reports^5^. This expenditure was attributed to processing only, exclusive of actual quality improvement activities^5^. Despite an extensive EMR in nearly all hospitals in the USA, few quality measures that must be reported are based on information that can be collected from EMRs electronically^36^. In an era of spiralling healthcare costs, there is a pressing need to re-evaluate how quality is assessed and how to leverage EMRs to efficiently monitor the quality of clinical care.

National programmes are valuable for interhospital comparisons but are limited because they are expensive, have long lag times before QA analyses are available for review, and, despite the expense of data collection, only partially risk adjust. Labelling outcomes as risk adjusted can be misleading if that risk adjustment is incomplete. Many factors can influence complication risks but only a few are typically incorporated in risk adjustment models. For example, poor nutritional status is commonly incorporated in risk adjustment models but how many previous operations a patient may have had is not, despite the latter being a major predictor of adverse outcomes. To avoid a false sense of security that follows from labelling a QA model as risk adjusted, risk adjustment in the system described in this study was not pursued. However, the Bayesian approach could be modified to a regression-based system that can accommodate risk adjustment^2,25,27,37^. Alternatively, if the risk profile for patients in a health system changes, complication rate thresholds for QA review can be changed to account for higher- or lower-risk patients.

The currently proposed QA system requires determination of acceptable surgical complication threshold rates and this remains challenging without consensus benchmarks in the literature. The long-run mean complication rate was used as a reasonable, data-driven internal standard, but health systems may set more ambitious thresholds (for example, below the institutional mean or aligned with external benchmarks) as performance improves, with the model recalibrating naturally to the updated target. In the present study, yearly data were sampled to mimic periodic review of complication data and for model development. In practise, once the statistical models are in place, they can be updated as frequently as data become available from the EMR.

The analytical approach shown in this article can be adopted to near-real-time complication monitoring in health systems that have integrated EMRs. Complications were identified from diagnostic codes appearing in encounters. Although coding may be somewhat delayed after hospital discharge, it generally appears in the EMR almost immediately following outpatient visits. Most of the complications assessed appeared in the outpatient record. Searches for these codes can be performed as frequently as the EMR is updated to also update the Bayesian outcomes monitoring model (updates are available for analysis biweekly in the study health system).

The proposed Bayesian monitoring system facilitates integration into existing EMR environments with minimal additional infrastructure. It requires an EMR capable of automated querying of structured fields (for example, procedure and diagnosis codes via CPT and ICD-10) on a regular schedule (daily to biweekly), which is feasible in most modern integrated systems (for example, Epic, Cerner) that support structured query language-like data extraction or application programming interfaces. The computation of posterior distributions can be performed on any conventional computer requiring only basic statistical software (for example, R or Python). For EMRs containing substantial unstructured text (for example, progress notes), the current model relies solely on structured diagnostic codes for simplicity and reproducibility; however, future extensions could incorporate natural language processing to identify complications from free text. Missing information (for example, uncoded complications or incomplete records) would likely bias estimates towards lower rates, similar to limitations in administrative databases; sensitivity analyses or imputation strategies could be explored in prospective implementations. The target population in the present study was defined as patients undergoing major colorectal procedures (CPT 44XXX series, excluding laparoscopic appendicectomy), identified via automated procedure code queries, with complications attributed if coded within 1 year after surgery. In clinical practice, alerts (for example, when posterior probability > 80% exceeds threshold) could be delivered following routine periodic updating of Bayesian models as new data become available to notify quality teams or surgeons about potential problems. For patients identified during subsequent chart review as potentially at highest risk (for example, those in flagged periods with multiple risk factors), suggested follow-up could include intensified surveillance (earlier clinic visits, imaging, or laboratory tests), enhanced preventive measures (for example, extended prophylaxis or mobilization protocols), or multidisciplinary discussion to mitigate risk.

Study limitations include the retrospective design (although mimicking prospective use), reliance on a database originally created for small bowel obstruction research (potentially biasing case mix), and the use of non-informative priors (sensitivity analyses are recommended in future work). Prospective implementation is needed to assess the impact of real-time alerts on practice and outcomes. This work showed the feasibility of collecting data periodically and updating models, resulting in refined estimates of the probability of postoperative complications occurring. The data set used was biased towards outpatient encounters such that some complications (for example, anastomotic leak) were diagnosed in this study many months after they occurred. This anomaly can be rectified by the inclusion of inpatient data. The analysis is also limited by the reliance on billing codes to identify complications, a weakness that can be overcome by natural language processing of the EMR.

This Bayesian, EMR-based monitoring system offers a cost-effective, statistically robust alternative to conventional QA approaches. By providing direct posterior probabilities of elevated complication risk without the distortions of frequentist repeated testing, it supports timely, data-driven interventions to enhance postoperative patient safety.

## Supplementary Material

zrag082_Supplementary_Data

## Data Availability

All data collected for this research are reported in the manuscript and accompanying supplementary material.
