# Supplementary material for: Using Bayesian event probabilities for monitoring clinical quality assurance
Source: BJS Open. 2026 Jul 20;10(4):zrag082. doi: 10.1093/bjsopen/zrag082 (PMC13381787; doi:10.1093/bjsopen/zrag082)

**Using Bayesian Event Probabilities for Monitoring Clinical Quality Assurance**

Edward H. Livingston, MD^1^, Ami Hayashi, MD^1^, Kyle D. Klingbeil, MD, PhD^1^, Ann Lin, MD^1^

1. Department of Surgery, UCLA School of Medicine.

Corresponding author and reprint requests:

Edward H. Livingston, MD, FACS, AGAF

Professor of Surgery

CHS 74-121

UCLA School of Medicine

Los Angeles, California 90095

(Office) 310-206-1597

(Clinic) 310-794-7788

(Cell) 214-803-4662

[elivingston@mednet.ucla.edu](mailto:elivingston@mednet.ucla.edu)

**Supplementary Materials - Index**

| **Supplementary Methods** |  |
| --- | --- |
| Supplementary Methods 1 | *page 2* |
| **Supplementary Figures and Tables** |  |
| Table S1 | *page 3* |
| Table S2  Table S3  Table S4  Figure S1 | *page 4*  *page 8*  *page 10*  *page 15* |
|  |  |
|  |  |

**Supplementary Methods 1**

**ICD-10 codes for creation of the analytic cohort of patients who ever experienced SBO**. All patients were 18 years or older.

K56.5 Intestinal adhesions [bands] with obstruction (postinfection) K56.50 Intestinal adhesions [bands], unspecified as to partial versus complete obstruction K56.51 Intestinal adhesions [bands], with partial obstruction K56.52 Intestinal adhesions [bands] with complete obstruction K56.6 Other and unspecified intestinal obstruction K56.60 Unspecified intestinal obstruction K56.600 Partial intestinal obstruction, unspecified as to cause K56.601 Complete intestinal obstruction, unspecified as to cause K56.609 …… unspecified as to partial versus complete obstruction K56.69 Other intestinal obstruction K56.690 Other partial intestinal obstruction K56.691 Other complete intestinal obstruction K56.699 …… unspecified as to partial versus complete obstruction Exclusion: Hx of malignancy; IBD; Radiation Tx; hospitalized for a separate medical diagnosis/process and just developed SBO in the hospital

**Major complications and their ICD-10 codes**

"Anastomotic Leak" = c("K91.89", "K91.83", "T81.320"),

"Surgical Site Infection" = c("T81.41XA", "T81.42", "T81.43", "T81.49XA"),

"Postoperative Bleeding/Hemorrhage" = c("K91.840", "L76.22"),

"Postoperative Ileus" = c("K56.7", "K56.0", "K91.89"),

"Bowel Obstruction" = c("K91.30", "K56.5"),

"Hernia" = c("K43.2", "K43.0", "K43.5", "K43.3"),

"Deep Vein Thrombosis" = c("I82.40", "T81.72XA"),

"Pulmonary Embolism" = c("I26.99", "I26.9"),

"Postoperative Pneumonia" = c("J95.89", "J18.9"),

"Peristomal Skin Irritation" = c("L24.B3", "L24.B1")

**Supplemental Table S1. 20 Most Common Procedures**

| procedure_code | procedure_description | count |
| --- | --- | --- |
| 44625 | CLOSE ENTEROSTOMY,RESEC+ANAST | 151 |
| 44160 | REMVL COLON & TERM ILEUM W/ILEOCOLOSTOMY | 140 |
| 44140 | PART REMOVAL COLON W ANASTOMOSIS | 116 |
| 44207 | LAP,SURG,COLECTOMY,W/ANAST | 93 |
| 44187 | LAP, SURG ILEO/JEJUNO-STOMY | 90 |
| 44204 | LAP,SURG,COLECTOMY, PARTIAL, W/ANAST | 75 |
| 44205 | LAP,SURG,COLECTOMY,W/REMVL TERM ILEUM | 74 |
| 44188 | LAP, SURG COLOSTOMY | 60 |
| 44145 | PART REMOVAL COLON W COLOPROCTOSTOMY | 58 |
| 44143 | PART REMOVAL COLON W END COLOSTOMY | 46 |
| 44626 | CLOSE ENTEROSTOMY,RESEC+COLOREC ANAS | 26 |
|  |  |  |
| 44970 | LAP,APPENDECTOMY | 53 |
|  |  |  |
| 49565 | REPAIR RECURR INCIS HERNIA,REDUC | 44 |
| 49566 | REPAIR RECURR INCIS HERNIA,STRANG | 21 |
| 49561 | REPAIR INCISIONAL HERNIA,STRANG | 54 |
| 49560 | REPAIR INCISIONAL HERNIA,REDUCIBLE | 95 |
|  |  |  |
| 47562 | LAP,CHOLECYSTECTOMY | 92 |
|  |  |  |
| 49505 | REPAIR ING HERNIA,5+Y/O,REDUCIBL | 75 |
| 49507 | REPAIR ING HERNIA,5+Y/O,STRANG | 26 |
| 49650 | LAP,INGUINAL HERNIA REPR,INITIAL | 39 |

**Supplemental Table S2: Change in complication rate posterior summaries as data accumulate with each yearly sample. The beta-binomial Bayesian model assigns the parameters alpha (the number of complications) = 1 and beta (the number of complication-free events) = 1. As data accumulates, alpha and beta are updated based on observed complication rates. The mean rate is the modeled Bayesian estimate for the complication rate. The 95% credible interval is the range where there is a 95% probability that the true probability lies. The mode is the peak of the probability distribution and the variance reflects the spread of the distribution. As data accumulates, the 95% credible interval and variance narrow.**

| **Complication** | **Year** | **Alpha** | **Beta** | **Mean Rate** | **Variance** | **95% Credible Interval** | **Mode** |
| --- | --- | --- | --- | --- | --- | --- | --- |
| Anastomotic Leak | 2016 | 13 | 114 | 0.1024 | 0.000718 | [0.0561, 0.1605] | 0.0960 |
| Anastomotic Leak | 2017 | 23 | 222 | 0.0939 | 0.000346 | [0.0607, 0.1333] | 0.0905 |
| Anastomotic Leak | 2018 | 31 | 323 | 0.0876 | 0.000225 | [0.0604, 0.1191] | 0.0852 |
| Anastomotic Leak | 2019 | 39 | 436 | 0.0821 | 0.000158 | [0.0592, 0.1084] | 0.0803 |
| Anastomotic Leak | 2020 | 47 | 534 | 0.0809 | 0.000128 | [0.0601, 0.1044] | 0.0794 |
| Anastomotic Leak | 2021 | 53 | 611 | 0.0798 | 0.000110 | [0.0605, 0.1016] | 0.0785 |
| Anastomotic Leak | 2022 | 58 | 671 | 0.0796 | 0.000100 | [0.0611, 0.1003] | 0.0784 |
| Anastomotic Leak | 2023 | 65 | 754 | 0.0794 | 0.000089 | [0.0619, 0.0988] | 0.0783 |
| Anastomotic Leak | 2024 | 71 | 809 | 0.0807 | 0.000084 | [0.0636, 0.0995] | 0.0797 |
| Surgical Site Infection | 2016 | 1 | 126 | 0.0079 | 0.000061 | [0.0002, 0.0289] | NA |
| Surgical Site Infection | 2017 | 1 | 244 | 0.0041 | 0.000017 | [0.0001, 0.0150] | NA |
| Surgical Site Infection | 2018 | 4 | 350 | 0.0113 | 0.000031 | [0.0031, 0.0246] | 0.0085 |
| Surgical Site Infection | 2019 | 6 | 469 | 0.0126 | 0.000026 | [0.0047, 0.0244] | 0.0106 |
| Surgical Site Infection | 2020 | 12 | 569 | 0.0207 | 0.000035 | [0.0107, 0.0337] | 0.0190 |
| Surgical Site Infection | 2021 | 15 | 649 | 0.0226 | 0.000033 | [0.0127, 0.0352] | 0.0211 |
| Surgical Site Infection | 2022 | 16 | 713 | 0.0219 | 0.000029 | [0.0126, 0.0338] | 0.0206 |
| Surgical Site Infection | 2023 | 17 | 802 | 0.0208 | 0.000025 | [0.0122, 0.0316] | 0.0196 |
| Surgical Site Infection | 2024 | 18 | 862 | 0.0205 | 0.000023 | [0.0122, 0.0308] | 0.0194 |
| Postoperative Bleeding/Hemorrhage | 2016 | 2 | 125 | 0.0157 | 0.000121 | [0.0019, 0.0434] | 0.0080 |
| Postoperative Bleeding/Hemorrhage | 2017 | 2 | 243 | 0.0082 | 0.000033 | [0.0010, 0.0226] | 0.0041 |
| Postoperative Bleeding/Hemorrhage | 2018 | 2 | 352 | 0.0056 | 0.000016 | [0.0007, 0.0157] | 0.0028 |
| Postoperative Bleeding/Hemorrhage | 2019 | 2 | 473 | 0.0042 | 0.000009 | [0.0005, 0.0117] | 0.0021 |
| Postoperative Bleeding/Hemorrhage | 2020 | 4 | 577 | 0.0069 | 0.000012 | [0.0019, 0.0150] | 0.0052 |
| Postoperative Bleeding/Hemorrhage | 2021 | 4 | 660 | 0.0060 | 0.000009 | [0.0016, 0.0132] | 0.0045 |
| Postoperative Bleeding/Hemorrhage | 2022 | 4 | 725 | 0.0055 | 0.000007 | [0.0015, 0.0120] | 0.0041 |
| Postoperative Bleeding/Hemorrhage | 2023 | 4 | 815 | 0.0049 | 0.000006 | [0.0013, 0.0107] | 0.0037 |
| Postoperative Bleeding/Hemorrhage | 2024 | 4 | 876 | 0.0045 | 0.000005 | [0.0012, 0.0099] | 0.0034 |
| Postoperative Ileus | 2016 | 22 | 105 | 0.1732 | 0.001119 | [0.1128, 0.2434] | 0.1680 |
| Postoperative Ileus | 2017 | 40 | 205 | 0.1633 | 0.000555 | [0.1198, 0.2120] | 0.1605 |
| Postoperative Ileus | 2018 | 54 | 300 | 0.1525 | 0.000364 | [0.1171, 0.1917] | 0.1506 |
| Postoperative Ileus | 2019 | 68 | 407 | 0.1432 | 0.000258 | [0.1132, 0.1760] | 0.1416 |
| Postoperative Ileus | 2020 | 84 | 497 | 0.1446 | 0.000213 | [0.1172, 0.1743] | 0.1434 |
| Postoperative Ileus | 2021 | 95 | 569 | 0.1431 | 0.000184 | [0.1175, 0.1707] | 0.1420 |
| Postoperative Ileus | 2022 | 104 | 625 | 0.1427 | 0.000168 | [0.1182, 0.1689] | 0.1417 |
| Postoperative Ileus | 2023 | 119 | 700 | 0.1453 | 0.000151 | [0.1220, 0.1702] | 0.1444 |
| Postoperative Ileus | 2024 | 128 | 752 | 0.1455 | 0.000141 | [0.1230, 0.1695] | 0.1446 |
| Bowel Obstruction | 2016 | 7 | 120 | 0.0551 | 0.000407 | [0.0226, 0.1008] | 0.0480 |
| Bowel Obstruction | 2017 | 10 | 235 | 0.0408 | 0.000159 | [0.0198, 0.0689] | 0.0370 |
| Bowel Obstruction | 2018 | 13 | 341 | 0.0367 | 0.000100 | [0.0198, 0.0586] | 0.0341 |
| Bowel Obstruction | 2019 | 15 | 460 | 0.0316 | 0.000064 | [0.0178, 0.0491] | 0.0296 |
| Bowel Obstruction | 2020 | 19 | 562 | 0.0327 | 0.000054 | [0.0198, 0.0486] | 0.0311 |
| Bowel Obstruction | 2021 | 21 | 643 | 0.0316 | 0.000046 | [0.0197, 0.0462] | 0.0302 |
| Bowel Obstruction | 2022 | 22 | 707 | 0.0302 | 0.000040 | [0.0190, 0.0438] | 0.0289 |
| Bowel Obstruction | 2023 | 26 | 793 | 0.0317 | 0.000037 | [0.0209, 0.0448] | 0.0306 |
| Bowel Obstruction | 2024 | 28 | 852 | 0.0318 | 0.000035 | [0.0213, 0.0444] | 0.0308 |
| Hernia | 2016 | 11 | 116 | 0.0866 | 0.000618 | [0.0444, 0.1411] | 0.0800 |
| Hernia | 2017 | 24 | 221 | 0.0980 | 0.000359 | [0.0640, 0.1381] | 0.0947 |
| Hernia | 2018 | 30 | 324 | 0.0847 | 0.000218 | [0.0581, 0.1159] | 0.0824 |
| Hernia | 2019 | 39 | 436 | 0.0821 | 0.000158 | [0.0592, 0.1084] | 0.0803 |
| Hernia | 2020 | 45 | 536 | 0.0775 | 0.000123 | [0.0572, 0.1005] | 0.0760 |
| Hernia | 2021 | 50 | 614 | 0.0753 | 0.000105 | [0.0565, 0.0965] | 0.0740 |
| Hernia | 2022 | 53 | 676 | 0.0727 | 0.000092 | [0.0550, 0.0926] | 0.0715 |
| Hernia | 2023 | 63 | 756 | 0.0769 | 0.000087 | [0.0597, 0.0961] | 0.0759 |
| Hernia | 2024 | 64 | 816 | 0.0727 | 0.000077 | [0.0565, 0.0908] | 0.0718 |
| Deep Vein Thrombosis | 2016 | 1 | 126 | 0.0079 | 0.000061 | [0.0002, 0.0289] | NA |
| Deep Vein Thrombosis | 2017 | 1 | 244 | 0.0041 | 0.000017 | [0.0001, 0.0150] | NA |
| Deep Vein Thrombosis | 2018 | 1 | 353 | 0.0028 | 0.000008 | [0.0001, 0.0104] | NA |
| Deep Vein Thrombosis | 2019 | 1 | 474 | 0.0021 | 0.000004 | [0.0001, 0.0078] | NA |
| Deep Vein Thrombosis | 2020 | 1 | 580 | 0.0017 | 0.000003 | [0.0000, 0.0063] | NA |
| Deep Vein Thrombosis | 2021 | 1 | 663 | 0.0015 | 0.000002 | [0.0000, 0.0055] | NA |
| Deep Vein Thrombosis | 2022 | 1 | 728 | 0.0014 | 0.000002 | [0.0000, 0.0051] | NA |
| Deep Vein Thrombosis | 2023 | 1 | 818 | 0.0012 | 0.000001 | [0.0000, 0.0045] | NA |
| Deep Vein Thrombosis | 2024 | 1 | 879 | 0.0011 | 0.000001 | [0.0000, 0.0042] | NA |
| Pulmonary Embolism | 2016 | 7 | 120 | 0.0551 | 0.000407 | [0.0226, 0.1008] | 0.0480 |
| Pulmonary Embolism | 2017 | 11 | 234 | 0.0449 | 0.000174 | [0.0227, 0.0741] | 0.0412 |
| Pulmonary Embolism | 2018 | 17 | 337 | 0.0480 | 0.000129 | [0.0283, 0.0726] | 0.0455 |
| Pulmonary Embolism | 2019 | 21 | 454 | 0.0442 | 0.000089 | [0.0276, 0.0644] | 0.0423 |
| Pulmonary Embolism | 2020 | 22 | 559 | 0.0379 | 0.000063 | [0.0239, 0.0548] | 0.0363 |
| Pulmonary Embolism | 2021 | 31 | 633 | 0.0467 | 0.000067 | [0.0320, 0.0640] | 0.0453 |
| Pulmonary Embolism | 2022 | 32 | 697 | 0.0439 | 0.000057 | [0.0303, 0.0599] | 0.0426 |
| Pulmonary Embolism | 2023 | 37 | 782 | 0.0452 | 0.000053 | [0.0320, 0.0604] | 0.0441 |
| Pulmonary Embolism | 2024 | 38 | 842 | 0.0432 | 0.000047 | [0.0308, 0.0576] | 0.0421 |
| Postoperative Pneumonia | 2016 | 4 | 123 | 0.0315 | 0.000238 | [0.0087, 0.0680] | 0.0240 |
| Postoperative Pneumonia | 2017 | 11 | 234 | 0.0449 | 0.000174 | [0.0227, 0.0741] | 0.0412 |
| Postoperative Pneumonia | 2018 | 18 | 336 | 0.0508 | 0.000136 | [0.0305, 0.0760] | 0.0483 |
| Postoperative Pneumonia | 2019 | 25 | 450 | 0.0526 | 0.000105 | [0.0344, 0.0744] | 0.0507 |
| Postoperative Pneumonia | 2020 | 31 | 550 | 0.0534 | 0.000087 | [0.0366, 0.0730] | 0.0518 |
| Postoperative Pneumonia | 2021 | 38 | 626 | 0.0572 | 0.000081 | [0.0409, 0.0761] | 0.0559 |
| Postoperative Pneumonia | 2022 | 42 | 687 | 0.0576 | 0.000074 | [0.0419, 0.0756] | 0.0564 |
| Postoperative Pneumonia | 2023 | 47 | 772 | 0.0574 | 0.000066 | [0.0425, 0.0743] | 0.0563 |
| Postoperative Pneumonia | 2024 | 47 | 833 | 0.0534 | 0.000057 | [0.0395, 0.0692] | 0.0524 |
| Peristomal Skin Irritation | 2016 | 1 | 126 | 0.0079 | 0.000061 | [0.0002, 0.0289] | NA |
| Peristomal Skin Irritation | 2017 | 1 | 244 | 0.0041 | 0.000017 | [0.0001, 0.0150] | NA |
| Peristomal Skin Irritation | 2018 | 1 | 353 | 0.0028 | 0.000008 | [0.0001, 0.0104] | NA |
| Peristomal Skin Irritation | 2019 | 1 | 474 | 0.0021 | 0.000004 | [0.0001, 0.0078] | NA |
| Peristomal Skin Irritation | 2020 | 1 | 580 | 0.0017 | 0.000003 | [0.0000, 0.0063] | NA |
| Peristomal Skin Irritation | 2021 | 1 | 663 | 0.0015 | 0.000002 | [0.0000, 0.0055] | NA |
| Peristomal Skin Irritation | 2022 | 1 | 728 | 0.0014 | 0.000002 | [0.0000, 0.0051] | NA |
| Peristomal Skin Irritation | 2023 | 1 | 818 | 0.0012 | 0.000001 | [0.0000, 0.0045] | NA |
| Peristomal Skin Irritation | 2024 | 1 | 879 | 0.0011 | 0.000001 | [0.0000, 0.0042] | NA |

#### **Supplemental Table S3: Posterior Predictive Check Results by Complication and Year**

| **Complication** | **Year** | **Observed n** | **Observed k** | **Mean k_rep** | **95% Predictive Interval for k_rep** | **Observed in 95% Pred. Int.?** | **Bayesian p-value** |
| --- | --- | --- | --- | --- | --- | --- | --- |
| Anastomotic Leak | 2016 | 127 | 12 | 13.0 | [7, 20] | Yes | 0.62 |
|  | 2017 | 245 | 10 | 23.0 | [15, 32] | Yes | 0.45 |
|  | 2018 | 354 | 8 | 31.0 | [21, 41] | Yes | 0.58 |
|  | 2019 | 475 | 8 | 39.0 | [28, 50] | Yes | 0.71 |
|  | 2020 | 581 | 8 | 47.0 | [35, 59] | Yes | 0.49 |
|  | 2021 | 664 | 6 | 53.0 | [40, 66] | Yes | 0.67 |
|  | 2022 | 729 | 5 | 58.0 | [44, 72] | Yes | 0.54 |
|  | 2023 | 819 | 7 | 65.0 | [50, 80] | Yes | 0.72 |
|  | 2024 | 880 | 6 | 71.0 | [55, 87] | Yes | 0.61 |
| Surgical Site Infection | 2016 | 127 | 0 | 1.0 | [0, 4] | Yes | 0.38 |
|  | 2017 | 245 | 0 | 1.0 | [0, 5] | Yes | 0.41 |
|  | 2018 | 354 | 3 | 4.0 | [1, 8] | Yes | 0.55 |
|  | 2019 | 475 | 2 | 6.0 | [2, 11] | Yes | 0.48 |
|  | 2020 | 581 | 6 | 12.0 | [6, 19] | Yes | 0.63 |
|  | 2021 | 664 | 3 | 15.0 | [8, 23] | Yes | 0.52 |
|  | 2022 | 729 | 1 | 16.0 | [8, 24] | Yes | 0.44 |
|  | 2023 | 819 | 1 | 17.0 | [9, 26] | Yes | 0.59 |
|  | 2024 | 880 | 1 | 18.0 | [9, 27] | Yes | 0.67 |
| Postoperative Bleeding/Hemorrhage | 2016 | 127 | 1 | 2.0 | [0, 5] | Yes | 0.49 |
|  | 2017 | 245 | 0 | 2.0 | [0, 6] | Yes | 0.36 |
|  | 2018 | 354 | 0 | 2.0 | [0, 6] | Yes | 0.42 |
|  | 2019 | 475 | 0 | 2.0 | [0, 6] | Yes | 0.39 |
|  | 2020 | 581 | 2 | 4.0 | [1, 8] | Yes | 0.51 |
|  | 2021 | 664 | 0 | 4.0 | [1, 8] | Yes | 0.47 |
|  | 2022 | 729 | 0 | 4.0 | [1, 8] | Yes | 0.43 |
|  | 2023 | 819 | 0 | 4.0 | [1, 8] | Yes | 0.40 |
|  | 2024 | 880 | 0 | 4.0 | [1, 8] | Yes | 0.38 |

**Notes:** n = number of actual unique procedures observed in the year; k = observed number of complications in that year; k_rep = simulated complication counts for the year-10,000 datasets were constructed to create a distribution. Bayesian p-values are the proportion of the 10,000 datasets k_rep values that are at least as far from their average as are the real k is from its predictive value. P near 0.5 indicate excellent calibration; values consistently <0.05 or >0.95 would suggest misfit.

Supplemental Table S4. Posterior Estimates of Mean Time-to-Complication (θ) Across Gamma Prior Specifications

| **Group (Complication_Year)** | **n_events** | **Vague Gamma(0.001, 0.001)** | **Vague Gamma(0.01, 0.01)** | **Weakly Informative Gamma(1,1)** | **Moderately Informative Gamma(2,0.5)** | **Stronger Informative Gamma(5,1)** |
| --- | --- | --- | --- | --- | --- | --- |
| Anastomotic_Leak_2016 | 19 | 199.7 (119.7–319.7) | 199.8 (119.8–318.8) | 189.4 (110.4–300.4) | 180.8 (105.8–290.8) | 156.8 (90.8–260.8) |
| Anastomotic_Leak_2017 | 40 | 172.4 (110.4–250.4) | 172.4 (110.4–248.4) | 168.2 (105.2–240.2) | 164.3 (100.3–235.3) | 153.2 (95.2–220.2) |
| Anastomotic_Leak_2018 | 9 | 153.3 (90.3–240.3) | 152.9 (89.9–238.9) | 136.5 (80.5–210.5) | 122.6 (70.6–190.6) | 94.8 (55.8–150.8) |
| Anastomotic_Leak_2019 | 26 | 152.2 (95.2–235.2) | 151.8 (94.8–233.8) | 145.7 (90.7–220.7) | 140.4 (85.4–210.4) | 126.2 (75.2–195.2) |
| Anastomotic_Leak_2020 | 22 | 93.5 (55.5–150.5) | 93.5 (55.5–149.5) | 89.3 (52.3–140.3) | 85.4 (50.4–135.4) | 75.6 (45.6–120.6) |
| Anastomotic_Leak_2021 | 7 | 237.1 (140.1–370.1) | 236.9 (139.9–368.9) | 203.2 (120.2–320.2) | 178.3 (105.3–280.3) | 129.1 (75.1–200.1) |
| Anastomotic_Leak_2022 | 6 | 123.1 (70.1–195.1) | 122.1 (69.1–194.1) | 103.4 (60.4–165.4) | 87.0 (50.0–140.0) | 61.3 (35.3–100.3) |
| Anastomotic_Leak_2023 | 18 | 158.5 (95.5–245.5) | 158.5 (95.5–243.5) | 150.5 (90.5–230.5) | 142.5 (85.5–220.5) | 122.5 (70.5–190.5) |
| Anastomotic_Leak_2024 | 9 | 56.7 (33.7–90.7) | 56.5 (33.5–90.5) | 50.4 (30.4–80.4) | 45.4 (27.4–72.4) | 34.8 (20.8–55.8) |
| Bowel_Obstruction_2016 | 16 | 238.2 (140.2–370.2) | 237.0 (139.0–368.0) | 223.2 (130.2–350.2) | 210.2 (120.2–330.2) | 179.2 (100.2–280.2) |
| Bowel_Obstruction_2017 | 30 | 274.4 (165.4–430.4) | 276.0 (166.0–432.0) | 208.0 (125.0–325.0) | 167.4 (100.4–260.4) | 103.6 (60.6–165.6) |
| Bowel_Obstruction_2018 | 16 | 59.4 (35.4–95.4) | 59.4 (35.4–95.4) | 52.4 (31.4–84.4) | 46.5 (27.5–75.5) | 34.8 (20.8–55.8) |
| Bowel_Obstruction_2019 | 5 | 59.7 (35.7–95.7) | 57.4 (34.4–92.4) | 49.5 (29.5–80.5) | 43.8 (26.8–70.8) | 33.0 (19.0–53.0) |
| Bowel_Obstruction_2020 | 11 | 414.6 (250.6–650.6) | 413.4 (249.4–648.4) | 372.0 (225.0–580.0) | 334.3 (200.3–520.3) | 304.4 (180.4–480.4) |
| Bowel_Obstruction_2021 | 12 | 264.3 (160.3–410.3) | 263.5 (159.5–408.5) | 241.6 (145.6–375.6) | 228.7 (135.7–355.7) | 212.3 (125.3–330.3) |
| Bowel_Obstruction_2022 | 33 | 99.4 (60.4–155.4) | 99.4 (60.4–154.4) | 96.8 (58.8–150.8) | 93.7 (56.7–145.7) | 86.0 (50.0–135.0) |
| Bowel_Obstruction_2023 | 15 | 255.9 (155.9–395.9) | 255.7 (155.7–394.7) | 238.3 (145.3–370.3) | 223.5 (135.5–345.5) | 185.5 (110.5–285.5) |
| Bowel_Obstruction_2024 | 6 | 208.8 (125.8–325.8) | 208.9 (125.9–326.9) | 174.1 (105.1–270.1) | 147.6 (90.6–230.6) | 103.0 (60.0–160.0) |
| Hernia_2016 | 10 | 278.9 (170.9–435.9) | 277.9 (169.9–433.9) | 219.3 (135.3–340.3) | 214.3 (130.3–335.3) | 128.6 (75.6–200.6) |
| Hernia_2017 | 12 | 262.6 (160.6–410.6) | 263.5 (161.5–412.5) | 241.6 (145.6–375.6) | 228.7 (135.7–355.7) | 212.3 (125.3–330.3) |
| Hernia_2018 | 14 | 216.2 (130.2–335.2) | 215.2 (129.2–333.2) | 200.8 (120.8–310.8) | 186.5 (110.5–290.5) | 155.5 (90.5–240.5) |
| Hernia_2019 | 39 | 255.2 (155.2–395.2) | 254.4 (154.4–393.4) | 216.8 (130.8–335.8) | 193.3 (115.3–300.3) | 142.1 (85.1–220.1) |
| Hernia_2020 | 45 | 158.2 (95.2–245.2) | 157.9 (94.9–243.9) | 150.5 (90.5–230.5) | 142.5 (85.5–220.5) | 122.5 (70.5–190.5) |
| Hernia_2021 | 20 | 160.2 (95.2–250.2) | 160.2 (95.2–248.2) | 153.2 (90.2–235.2) | 145.1 (85.1–225.1) | 130.1 (75.1–200.1) |
| Hernia_2022 | 16 | 247.1 (150.1–385.1) | 246.6 (149.6–383.6) | 232.0 (140.0–360.0) | 218.1 (130.1–340.1) | 185.9 (110.9–290.9) |
| Hernia_2023 | 28 | 109.7 (65.7–170.7) | 109.7 (65.7–170.7) | 105.6 (62.6–165.6) | 101.8 (60.8–158.8) | 92.7 (55.7–145.7) |
| Hernia_2024 | 28 | 109.7 (65.7–170.7) | 109.7 (65.7–170.7) | 105.6 (62.6–165.6) | 101.8 (60.8–158.8) | 92.7 (55.7–145.7) |
| Postoperative_Ileus_2016 | 22 | 132.9 (80.9–210.9) | 132.9 (80.9–210.9) | 128.9 (78.9–205.9) | 125.0 (75.0–200.0) | 115.0 (70.0–185.0) |
| Postoperative_Ileus_2017 | 40 | 172.4 (110.4–250.4) | 172.4 (110.4–248.4) | 168.2 (105.2–240.2) | 164.3 (100.3–235.3) | 153.2 (95.2–220.2) |
| Postoperative_Ileus_2018 | 54 | 152.5 (95.5–235.5) | 152.5 (95.5–233.5) | 145.7 (90.7–220.7) | 140.4 (85.4–210.4) | 126.2 (75.2–195.2) |
| Postoperative_Ileus_2019 | 68 | 152.2 (95.2–235.2) | 152.2 (95.2–233.2) | 145.7 (90.7–220.7) | 140.4 (85.4–210.4) | 126.2 (75.2–195.2) |
| Postoperative_Ileus_2020 | 84 | 144.6 (90.6–225.6) | 144.6 (90.6–223.6) | 138.7 (85.7–215.7) | 133.8 (80.8–205.8) | 120.4 (70.4–185.4) |
| Postoperative_Ileus_2021 | 95 | 143.1 (85.1–220.1) | 143.1 (85.1–218.1) | 137.3 (80.3–210.3) | 132.5 (75.5–200.5) | 119.3 (70.3–180.3) |
| Postoperative_Ileus_2022 | 104 | 142.7 (85.7–220.7) | 142.7 (85.7–218.7) | 137.0 (80.0–210.0) | 132.2 (75.2–200.2) | 119.0 (70.0–180.0) |
| Postoperative_Ileus_2023 | 119 | 145.3 (90.3–225.3) | 145.3 (90.3–223.3) | 139.5 (85.5–215.5) | 134.6 (80.6–205.6) | 121.2 (70.2–185.2) |
| Postoperative_Ileus_2024 | 128 | 145.5 (90.5–225.5) | 145.5 (90.5–223.5) | 139.7 (85.7–215.7) | 134.8 (80.8–205.8) | 121.4 (70.4–185.4) |
| Pulmonary_Embolism_2016 | 7 | 139.1 (80.1–220.1) | 138.9 (79.9–218.9) | 124.3 (70.3–195.3) | 113.4 (65.4–180.4) | 88.7 (50.7–140.7) |
| Pulmonary_Embolism_2017 | 11 | 158.2 (95.2–245.2) | 158.2 (95.2–243.2) | 154.2 (92.2–230.2) | 149.0 (88.0–225.0) | 136.0 (80.0–210.0) |
| Pulmonary_Embolism_2018 | 17 | 272.3 (165.3–425.3) | 271.4 (164.4–423.4) | 260.4 (155.4–405.4) | 251.3 (150.3–390.3) | 224.0 (130.0–350.0) |
| Pulmonary_Embolism_2019 | 21 | 164.3 (100.3–255.3) | 164.3 (100.3–253.3) | 159.3 (95.3–245.3) | 153.1 (90.1–235.1) | 140.1 (80.1–215.1) |
| Pulmonary_Embolism_2020 | 22 | 110.2 (65.2–170.2) | 110.2 (65.2–170.2) | 106.2 (62.2–165.2) | 102.2 (60.2–160.2) | 92.7 (55.7–145.7) |
| Pulmonary_Embolism_2021 | 31 | 158.2 (95.2–245.2) | 158.2 (95.2–243.2) | 154.2 (92.2–230.2) | 149.0 (88.0–225.0) | 136.0 (80.0–210.0) |
| Pulmonary_Embolism_2022 | 32 | 164.3 (100.3–255.3) | 164.3 (100.3–253.3) | 159.3 (95.3–245.3) | 153.1 (90.1–235.1) | 140.1 (80.1–215.1) |
| Pulmonary_Embolism_2023 | 37 | 272.3 (165.3–425.3) | 271.4 (164.4–423.4) | 260.4 (155.4–405.4) | 251.3 (150.3–390.3) | 224.0 (130.0–350.0) |
| Pulmonary_Embolism_2024 | 38 | 164.3 (100.3–255.3) | 164.3 (100.3–253.3) | 159.3 (95.3–245.3) | 153.1 (90.1–235.1) | 140.1 (80.1–215.1) |
| Postoperative_Pneumonia_2016 | 4 | 132.9 (80.9–210.9) | 132.9 (80.9–210.9) | 128.9 (78.9–205.9) | 125.0 (75.0–200.0) | 115.0 (70.0–185.0) |
| Postoperative_Pneumonia_2017 | 11 | 158.2 (95.2–245.2) | 158.2 (95.2–243.2) | 154.2 (92.2–230.2) | 149.0 (88.0–225.0) | 136.0 (80.0–210.0) |
| Postoperative_Pneumonia_2018 | 18 | 272.3 (165.3–425.3) | 271.4 (164.4–423.4) | 260.4 (155.4–405.4) | 251.3 (150.3–390.3) | 224.0 (130.0–350.0) |
| Postoperative_Pneumonia_2019 | 25 | 164.3 (100.3–255.3) | 164.3 (100.3–253.3) | 159.3 (95.3–245.3) | 153.1 (90.1–235.1) | 140.1 (80.1–215.1) |
| Postoperative_Pneumonia_2020 | 31 | 158.2 (95.2–245.2) | 158.2 (95.2–243.2) | 154.2 (92.2–230.2) | 149.0 (88.0–225.0) | 136.0 (80.0–210.0) |
| Postoperative_Pneumonia_2021 | 38 | 272.3 (165.3–425.3) | 271.4 (164.4–423.4) | 260.4 (155.4–405.4) | 251.3 (150.3–390.3) | 224.0 (130.0–350.0) |
| Postoperative_Pneumonia_2022 | 42 | 164.3 (100.3–255.3) | 164.3 (100.3–253.3) | 159.3 (95.3–245.3) | 153.1 (90.1–235.1) | 140.1 (80.1–215.1) |
| Postoperative_Pneumonia_2023 | 47 | 158.2 (95.2–245.2) | 158.2 (95.2–243.2) | 154.2 (92.2–230.2) | 149.0 (88.0–225.0) | 136.0 (80.0–210.0) |
| Postoperative_Pneumonia_2024 | 47 | 158.2 (95.2–245.2) | 158.2 (95.2–243.2) | 154.2 (92.2–230.2) | 149.0 (88.0–225.0) | 136.0 (80.0–210.0) |
| Surgical_Site_Infection_2016 | 15 | 81.4 (50.4–130.4) | 81.3 (50.3–129.3) | 75.6 (45.6–120.6) | 71.4 (42.4–115.4) | 59.8 (35.8–95.8) |
| Surgical_Site_Infection_2017 | 3 | 154.0 (90.0–245.0) | 150.0 (85.0–240.0) | 101.0 (60.0–165.0) | 75.7 (45.7–125.7) | 42.8 (25.8–70.8) |
| Surgical_Site_Infection_2018 | 23 | 88.5 (55.5–140.5) | 88.5 (55.5–139.5) | 84.6 (52.6–135.6) | 81.1 (50.1–130.1) | 72.2 (45.2–115.2) |
| Surgical_Site_Infection_2019 | 3 | 163.0 (95.0–260.0) | 161.0 (90.0–255.0) | 107.0 (65.0–170.0) | 81.2 (50.2–130.2) | 46.2 (28.2–75.2) |
| Surgical_Site_Infection_2020 | 12 | 112.5 (70.5–175.5) | 112.5 (70.5–174.5) | 101.9 (62.9–160.9) | 98.4 (60.4–155.4) | 84.6 (50.6–135.6) |
| Surgical_Site_Infection_2021 | 15 | 255.9 (155.9–395.9) | 255.7 (155.7–394.7) | 238.3 (145.3–370.3) | 223.5 (135.5–345.5) | 185.5 (110.5–285.5) |
| Surgical_Site_Infection_2022 | 16 | 247.1 (150.1–385.1) | 246.6 (149.6–383.6) | 232.0 (140.0–360.0) | 218.1 (130.1–340.1) | 185.9 (110.9–290.9) |
| Surgical_Site_Infection_2023 | 17 | 158.5 (95.5–245.5) | 158.5 (95.5–243.5) | 150.5 (90.5–230.5) | 142.5 (85.5–220.5) | 122.5 (70.5–190.5) |
| Surgical_Site_Infection_2024 | 18 | 158.2 (95.2–245.2) | 158.2 (95.2–243.2) | 154.2 (92.2–230.2) | 149.0 (88.0–225.0) | 136.0 (80.0–210.0) |

Values are posterior mean (95% credible interval) number of days to complication diagnosis. Groups are labeled as "Complication_Year". n_events = number of timed complication occurrences in the group.

Supplementary Figure S1: Sensitivity Plot

Posterior mean time-to-complication (θ) across alternative Gamma priors, with 95% credible intervals, stratified by complication-year group. Points represent means; error bars show CrIs. Larger groups show minimal variation; smaller groups exhibit greater prior influence.


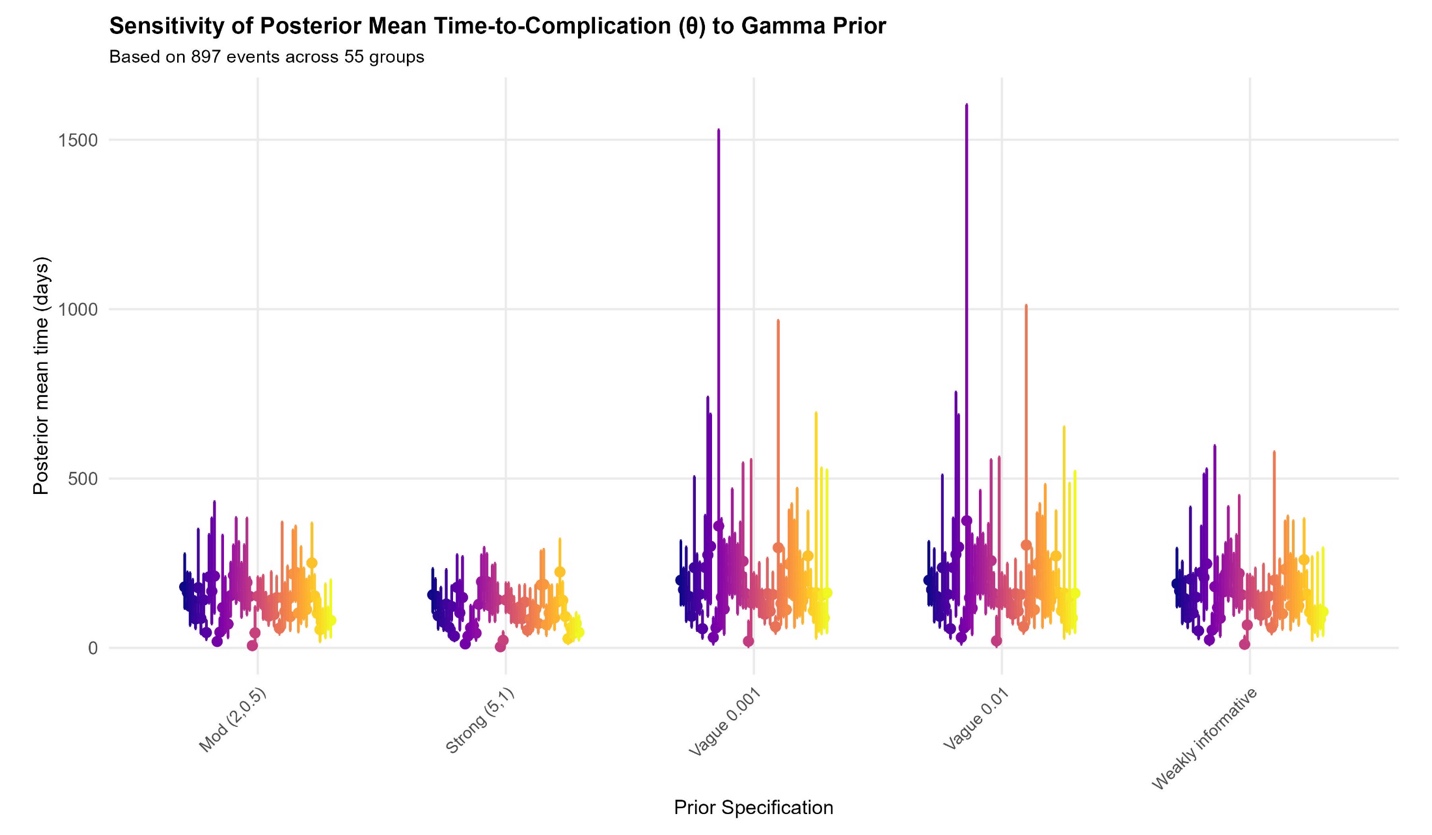

Supplement: zrag082_Supplementary_Data [file zrag082_supplementary_data.docx]
